# Acute Kidney Injury-to-Chronic Kidney Disease Transition-Associated Macrophage Subtypes: Biological Functions and Intercellular Crosstalk

**DOI:** 10.3390/ijms27177910

**Published:** 2026-09-04

**Authors:** Shuai Jin, Chenrong Fu, Haoran Zhang, Yingying Ji, Qing Jiao, Peng Liu

**Affiliations:** 1 Department of Pharmacology, Shenyang Pharmaceutical University, 103 Wenhua Road, Shenhe District, Shenyang 110016, China; s13944622391@163.com (S.J.); fucr12866@foxmail.com (C.F.); haor0327@163.com (H.Z.); 16635400480@163.com (Y.J.); 2 Department of Clinical Pharmacy, Shenyang Pharmaceutical University, 103 Wenhua Road, Shenhe District, Shenyang 110016, China; 3 Joint International Research Laboratory of Intelligent Drug Delivery Systems, Ministry of Education, 103 Wenhua Road, Shenhe District, Shenyang 110016, China

**Keywords:** acute kidney injury, chronic kidney disease, kidney injury transition, macrophages

## Abstract

As a core organ responsible for metabolism and homeostatic regulation, the kidney performs physiological functions that encompass material excretion, fluid balance, electrolyte regulation, and endocrine control; and serves as a critical hub for maintaining the coordinated functioning of multiple organ systems. Kidney injury not only leads to disturbances in these core physiological functions but also generates systemic complications such as cardiovascular disease, hypertension, and diabetes mellitus through an “injury–inflammation–metabolic disorder” cascade. Epidemiologic studies and clinical statistics have revealed that acute kidney injury (AKI) may progress to chronic kidney disease (CKD) because of maladaptive repair, impaired regeneration, and other factors. During this process, macrophages—particularly certain functionally specialized macrophage subtypes—engage in complex intercellular communication with neighboring cells that include renal tubular epithelial cells, endothelial cells, fibroblasts, and platelets, thereby forming pathological signaling networks that collectively drive persistent inflammation and the progression of renal fibrosis. Macrophages thus play complex and dynamic dual regulatory roles throughout the initiation, progression, and repair of kidney injury, and their functional polarization and phenotypic transformation directly influence the pathological progression of kidney diseases. We herein aimed to elucidate the roles of AKI-to-CKD transition-associated macrophages, especially the subtypes with specialized functions in kidney diseases and to systematically review current research progress, thus to provide a reference for advancing basic research and clinical diagnostic and therapeutic strategies for kidney diseases.

## 1. Introduction

The kidney is a core organ that is responsible for the regulation of metabolism and the maintenance of internal homeostasis. Although acute kidney injury (AKI) and chronic kidney disease (CKD) are traditionally regarded as two distinct disease entities, with advances in epidemiologic studies and the accumulation of clinical statistical data, it has become evident that AKI may subsequently progress to CKD due to maladaptive repair, impaired regeneration, and other factors [1,2,3]. The latest data from the Global Burden of Disease Study 2023 reveal that the global disease burden of chronic kidney disease (CKD) has continued to increase over the past three decades. The number of adults living with CKD worldwide reached 788 million in 2023, nearly doubling from 378 million in 1990 and indicating a sustained expansion of the global CKD population. Meanwhile, the global age-standardized mortality rate of CKD increased by 6.1% over the same period, rising from 24.9 per 100,000 population in 1990 to 26.5 per 100,000 population in 2023. CKD therefore represents one of the few chronic diseases with a persistently growing mortality burden worldwide [4].

Renal fibrosis is the central pathological feature of CKD and is characterized by the abnormal proliferation and excessive accumulation of connective tissue. Renal fibrosis is not only a key pathological factor that drives CKD progression, but it is also the common pathological pathway by which CKD due to various kidney diseases (such as tubular nephropathy, interstitial nephropathy, and vascular nephropathy) advances to end-stage kidney disease [5]. Among various cellular components driving renal fibrosis development, macrophages serve as critical regulators of renal injury response and tissue repair [6]. Macrophages are immune cells derived from hematopoietic stem cells residing in the bone marrow and are widely distributed throughout various tissues and organs of the body. Macrophages participate in promoting tissue repair and regeneration, phagocytosing and clearing metabolic waste and regulating immune responses, thereby comprising an important component of the body’s immune defense system [7].

Accordingly, this review focuses on the pathological progression of renal injury and subsequent AKI-to-CKD transition, summarizes the functional characteristics of distinct macrophage subtypes throughout renal injury, progression, and repair imbalance, and further explores their potential translational and therapeutic implications for kidney diseases. The pre-clinical animal studies cited in this review are predominantly based on intrinsic renal AKI induced by ischemia–reperfusion injury, which further progresses to CKD. In terms of human-relevant research evidence, this manuscript mainly focuses on the pathological transition process of secondary AKI mediated by ischemia–reperfusion injury, toxins and inflammation. Pre-renal AKI, post-renal AKI, as well as diabetic nephropathy and hypertensive nephropathy without a prior history of AKI are not covered in the present review.

In this review, we performed literature searches in the PubMed and Web of Science databases, using keyword combinations: “acute kidney injury”, “chronic kidney disease”, “AKI-to-CKD transition”, “macrophage”, “single-cell RNA-seq”, “spatial transcriptomics”, “immunometabolism”. Inclusion criteria were as follows: original articles and peer-reviewed reviews focusing on macrophage heterogeneity, intercellular crosstalk and immunometabolism during AKI-to-CKD transition, both human and animal studies. Exclusion criteria were as follows: articles irrelevant to renal disease; conference abstracts, case reports. Information extracted from eligible publications included macrophage subsets, molecular signatures, spatial localization, immunometabolic features, cell-to-cell interaction and therapeutic findings.

Accumulating evidence has demonstrated that intrinsic functional heterogeneity and phenotypic plasticity of macrophages are critical determinants governing the severity of renal injury and the rate of progressive renal function loss [8].

## 2. Types of Kidney Injury and Transitional Mechanisms

Kidney injury can be classified into AKI and CKD. AKI is defined as a pathological condition that is characterized by the rapid decline or even complete loss of renal function within a short period due to various factors that include ischemia, toxins, drugs, and infections [9]. CKD is a progressive disease characterized by a persistent decline in renal function, with a history of kidney damage lasting for more than three months [10].

**Figure 1:**
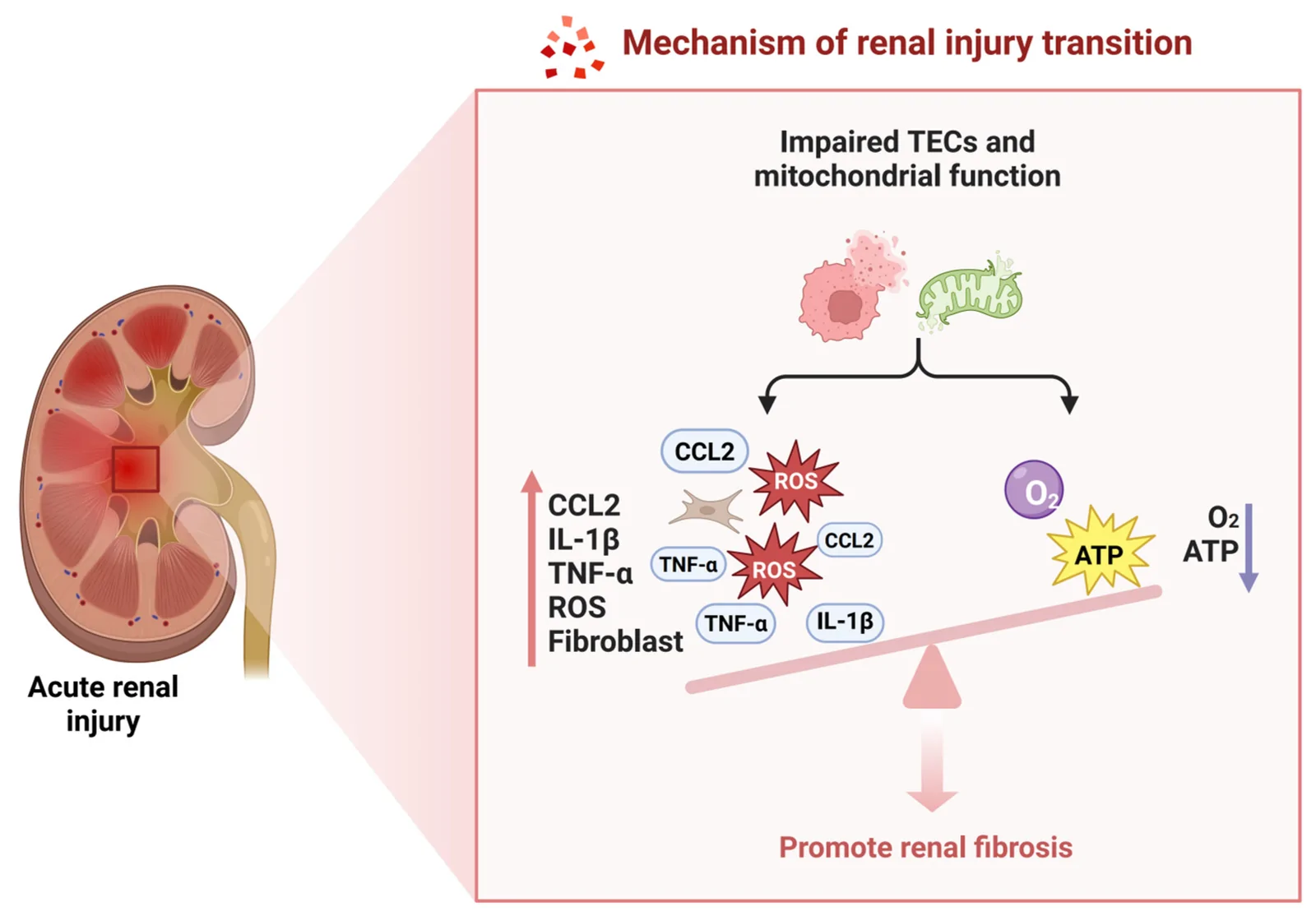
Mechanisms of kidney injury and transition. Damaged TECs with dysfunctional mitochondria trigger two harmful cascades: (1) release inflammatory mediators including CCL2, IL-1β, TNF-α and excess ROS, which activate fibroblasts and amplify inflammatory signals; (2) Mitochondrial damage reduces oxygen supply and ATP production, creating an energy imbalance. The combined pro-inflammatory, oxidative stress and metabolic dysfunction signals synergistically tip the balance to promote renal fibrosis, causing AKI and CKD. TECs: tubular epithelial cells; CCL2: C-C motif ligand 2; IL-1β: interleukin-1 beta; TNF-α: tumor necrosis factor-alpha; ROS: reactive oxygen species. Created in BioRender. Bucuo, J. (2026) https://BioRender.com/8r9nfem.

As a highly energy- and oxygen-consuming organ, the maintenance of normal kidney function is highly dependent upon adequate blood perfusion and oxygen supply. When factors such as shock, dehydration, and renal artery stenosis reduce renal blood flow, renal tubular epithelial cells (TECs) are the first to develop functional abnormalities due to insufficient energy and nutrient supply, thereby triggering AKI [11]. Following the onset of AKI, TECs and mitochondrial functions are impaired, with cells compensating by increasing oxygen consumption to maintain basic physiological activities; and this adaptive response further exacerbates hypoxia within the microenvironment of the renal tissue [12,13]. During the repair phase, mitochondrial structure and function remain abnormal, resulting in insufficient ATP production and an inability to provide adequate energy for TEC proliferation and repair. Injury repair is consequently hindered, and this promotes the transition from AKI to CKD [14,15,16]. Under stress conditions such as ischemia–hypoxia, toxin exposure, and mitochondrial dysfunction, the endogenous redox balance of the body is also disrupted, leading to the generation of a large amount of reactive oxygen species (ROS). ROS can directly induce fibroblast activation, accelerate abnormal extracellular matrix deposition, and promote the progression of renal fibrosis [17]; and ROS also activate inflammatory signaling pathways such as NF-κB and MAPK, which promotes the release of inflammatory mediators such as CCL2, TNF-α, and IL-1β and triggers sustained immune responses [18] (Figure 1).

As the central effector and regulatory cell type of the innate immune system, macrophages sense injury signals, are recruited to sites of injury, and are polarized into specific subtypes. These cells amplify immune responses via the secretion of inflammatory mediators and regulate cellular repair or fibrotic processes by interacting with TECs and fibroblasts [19]. Given the above context, this review summarizes and analyzes how various macrophage subsets participate in renal injury progression, establishing a theoretical basis for the development of targeted strategies to block sustained AKI-to-CKD transition.

## 3. Origins and Classification of Renal Macrophages

According to their ontogeny, renal macrophages can be broadly classified into two populations: kidney-resident macrophages (KRMs) and recruited macrophages (RMs). KRMs originate during early embryonic development [20], and their differentiation is independent of conventional hematopoietic stem cells and circulating monocytes; moreover, they possess the capacity to maintain population homeostasis through local self-renewal [21]. During the AKI-to-CKD transition, KRMs primarily function as a restorative macrophage subset that occupies roles in pathogen clearance, phagocytosis of apoptotic cells and tissue debris, maintenance of renal tubular structural integrity, and preservation of physiological homeostasis of the kidney [22,23].

RMs originate from monocytes that differentiated from bone marrow hematopoietic stem cells. During AKI, RMs recognize damage-associated molecular patterns (DAMPs) released by injured tissues and are selectively recruited to injury sites where they release inflammatory mediators such as IL-1β and TNF-α, amplify immune responses, and accentuate renal tubular injury. In the later stages of AKI, the prolonged maintenance of either pro-inflammatory phenotypes or aberrant restorative phenotypes accelerates the progression of renal interstitial fibrosis, making RMs one of the major driving factors in the transition from AKI to CKD.

## 4. Roles of Macrophages in the Kidney

### 4.1. Classical M1 and M2 Polarization Models and Their Limitations

The classical macrophage classification system that is currently accepted is based primarily on differences in activational status and functional specificity, and categorizes macrophages into two major functional subtypes: M1 macrophages (also known as classically activated macrophages) that are activated by interferon (IFN) and lipopolysaccharide (LPS) and mediate pro-inflammatory responses and pathogen clearance; and M2 macrophages (also known as alternatively activated macrophages) that are induced by interleukin-4 (IL-4) and possess anti-inflammatory, tissue repair, and immune homeostasis-maintaining actions [24]. Tissue-injury responses and healing processes are typically characterized by a transition from the M1 phenotype to the M2 phenotype [25].

While this binary classification has been instrumental in understanding macrophage biology, it is overly simplistic in the context of the acute kidney injury (AKI) to chronic kidney disease (CKD) transition. Recent advances, particularly single-cell RNA sequencing (scRNA-seq), have revealed that renal macrophages exhibit a continuous spectrum of phenotypes rather than discrete M1 or M2 states. For example, macrophages undergo dynamic transitions marked by changes in Ly6C expression, shifting from Ly6C(high) inflammatory monocytes to Ly6C(low) reparative macrophages [26]. Moreover, distinct subpopulations expressing markers such as Cx3cr1 and Trem2 have been identified, which do not fit neatly into the M1/M2 paradigm [27,28]. These transitional and specialized subsets contribute uniquely to the pathophysiology of AKI-to-CKD progression, mediating complex intercellular crosstalk and modulating the balance between inflammation resolution and fibrogenesis. Thus, the classical M1/M2 model, while foundational, fails to capture the heterogeneity and plasticity of macrophage responses in renal injury and repair, underscoring the need for more nuanced classification systems that integrate transcriptomic and functional data to better understand macrophage roles in kidney disease progression.

### 4.2. Pro-Inflammatory Macrophage Subtypes (M1-like) in Early AKI

In the initial phase of AKI, DAMPs, such as high-mobility group box 1 (HMGB1), activate resident and infiltrating macrophages, polarizing them toward a pro-inflammatory M1-like phenotype. These macrophages produce high levels of pro-inflammatory cytokines including TNF-α, IL-1β, and reactive oxygen species (ROS), which amplify the acute inflammatory response and recruit additional immune cells to the site of injury. The M1-like macrophages also engage in phagocytosis of necrotic tubular epithelial cells and cellular debris, a critical process for clearing damaged tissue and initiating repair. Furthermore, they secrete matrix metalloproteinases (MMPs) that degrade extracellular matrix components, facilitating tissue remodeling. However, excessive or prolonged activation of M1-like macrophages can exacerbate tubular epithelial cell apoptosis and disrupt the microvascular integrity, leading to capillary leakage and further renal damage. This maladaptive inflammatory milieu contributes to the severity of AKI and sets the stage for maladaptive repair mechanisms that promote fibrosis and CKD development. The balance and timely resolution of M1-like macrophage activity are therefore crucial determinants of renal recovery versus progression to chronic injury [29,30].

### 4.3. Reparative and Pro-Fibrotic Macrophage Subtypes (M2-like) in CKD Stage Transition

As kidney injury persists, macrophages gradually shift toward an M2-like phenotype characterized by the secretion of anti-inflammatory cytokines such as IL-10 and profibrotic mediators including TGF-β and platelet-derived growth factor (PDGF). These M2-like macrophages promote wound healing by stimulating fibroblast activation and extracellular matrix deposition, processes essential for tissue repair but which, when dysregulated, lead to renal interstitial fibrosis and irreversible functional decline. Notably, transitional macrophage subtypes expressing markers such as ApoE and Glycoprotein non-metastatic melanoma protein b (Gpnmb) have been identified as pivotal players in the AKI-to-CKD transition [31]. These subsets sustain the production of fibrogenic factors, perpetuating chronic inflammation and fibrosis. Their persistence correlates with progressive renal scarring and loss of nephron function. The identification of these transitional macrophage populations highlights the complexity of macrophage-mediated crosstalk in the fibrotic microenvironment and suggests potential therapeutic targets to modulate macrophage phenotypes and interrupt the progression from AKI to CKD [32].

### 4.4. Metabolic Reprogramming of Macrophages

The concept of metabolic reprogramming can be traced back to the pioneering discovery of the Warburg effect, which revealed that tumor cells preferentially obtain energy through glycolysis rather than by relying on the highly efficient energy-producing process of mitochondrial oxidative phosphorylation—even under conditions of adequate oxygen availability [33]. With advances in research this concept has acquired a broader definition, and now refers to the biological process by which cells systematically remodel and functionally redirect their intrinsic metabolic pathways under pathological conditions or in specific physiological contexts in order to adapt to the surrounding microenvironment and meet the core demands of rapid proliferation [34].

During macrophage polarization, M1 macrophages primarily rely on aerobic glycolysis as their main mode of energy metabolism, whereas M2 macrophages depend on oxidative phosphorylation and fatty acid oxidation to meet their energy requirements [35,36]. Numerous studies have confirmed that macrophages undergo metabolic reprogramming after stimulation by pro-inflammatory signals such as LPS and IFN-γ. The TLR4-mediated downstream NF-κB–STAT1 signaling cascade then activates hypoxia-inducible factor 1 (HIF-1) that upregulates glucose transporter 1 (GLUT1), hexokinase 2 (HK2), lactate dehydrogenase, and other key glycolytic enzymes—thereby significantly increasing overall glycolytic flux [37,38,39]. The pro-inflammatory microenvironment evokes elevated expression of inducible nitric oxide synthase (iNOS) in macrophages, and iNOS catalyzes the generation of large amounts of nitric oxide (NO) from L-arginine. NO directly binds to complexes of the mitochondrial electron transport chain, inhibits normal respiratory chain function, impedes oxidative phosphorylation, and blocks the tricarboxylic acid cycle pathway. This process subsequently promotes the abnormal accumulation of metabolic intermediates such as succinate and lactate, further inhibiting the ubiquitin-mediated degradation of HIF-1 protein and thus establishing a positive-feedback regulatory pathway. Macrophages quickly and ultimately shift from the oxidative phosphorylation-dependent metabolic pattern of the resting state to aerobic glycolysis, which maintains the M1 pro-inflammatory phenotype and reinforces the pro-inflammatory state of the macrophages [40,41,42].

Ding et al. [43] established a mouse renal ischemia–reperfusion injury model and demonstrated that HIF-1-regulated macrophage metabolic reprogramming was an important molecular mechanism driving the evolution from AKI to CKD. An abnormal inflammatory microenvironment is established following kidney injury, and this significantly upregulates HIF-1 expression in macrophages and subsequently induces the remodeling of macrophage glucose metabolism. This action promotes the extensive recruitment and infiltration of pro-inflammatory macrophages, continuously amplifies local renal inflammatory responses, and exacerbates renal tissue injury and fibrosis. Therefore, targeted inhibition of HIF-1 or specific abrogation of macrophage glycolytic pathways may represent highly promising and novel therapeutic targets for preventing and treating the transition from AKI to CKD.

## 5. Macrophage Subtypes with Specialized Functions Observed During the AKI-to-CKD Transition

During the transition from AKI to CKD, macrophages do not function independently. Instead, macrophages engage in complex intercellular crosstalk with neighboring cells comprising renal tubular epithelial cells, endothelial cells, fibroblasts, and platelets—thereby forming pathological signaling networks that collectively drive persistent inflammation and the progression of renal fibrosis. Injured renal tubules release chemokines and damage signals that recruit and activate macrophages, and activated macrophages further exacerbate tubular injury and abnormal fibroblast activation through the secretion of pro-inflammatory and pro-fibrotic factors. Interactions among macrophages, endothelial cells, and platelets ultimately regulate local microvascular injury and the homeostasis of the inflammatory microenvironment. Collectively, these processes constitute a multicellular, interaction-driven pathological circuit of renal fibrosis that represents a key mechanism that underlies the irreversible transition from AKI to CKD [44,45]. Therefore, the traditional M1/M2 binary paradigm cannot fully recapitulate macrophage heterogeneity during renal injury. In recent years, an increasing number of studies have employed single-cell and spatial transcriptomics to identify novel macrophage subpopulations.

### 5.1. Extracellular Matrix Remodeling-Associated Macrophages (EAMs)

The extracellular matrix (ECM) is an important component of the cellular microenvironment, provides structural support and physical barriers, mediates cellular adhesion and signal transduction, and participates in tissue repair and regeneration [46]. Clinical studies have confirmed that abnormal ECM deposition accompanies a variety of kidney diseases. Collagen, the most fundamental structural component of the ECM, undergoes metabolic imbalance within the renal interstitial matrix and thus directly contributes to the aggravation of renal interstitial fibrosis. As kidney disease progresses and large numbers of nephrons are damaged, the body’s capacity for collagen metabolism declines progressively. Additional studies revealed the concentrations of collagen degradation markers in patients with intermediate-stage kidney disease were significantly higher relative to those in patients with advanced-stage disease [47,48]. The aforementioned observations of abnormal ECM deposition and reduced collagen metabolism collectively indicate that regulating dynamic equilibrium of the ECM is of critical importance in repair of kidney injury.

Using integrated single-cell RNA sequencing and spatial transcriptomic technologies, Zhang et al. [49] characterized the macrophage lineage during the AKI-to-CKD transition, and demonstrated that restorative macrophages were highly expressed during the early stages of kidney injury and that they participated in tissue repair. However, their proportions declined significantly at later stages, and exhaustion of their functions led to impaired renal repair, creating conditions favorable for the development of renal fibrosis. In contrast, the expression of pro-inflammatory macrophages gradually increased as kidney injury progressed, exacerbating tissue damage by the maintenance of an inflammatory microenvironment. Among these populations, EAMs participate in tissue repair during the early phase of injury by promoting ECM deposition. At later stages, however, they undergo a transition toward a pro-inflammatory phenotype and interact with fibroblasts through the IGF1–IGF1R signaling axis, thereby promoting the development of renal fibrosis. Since investigators have confirmed that fibroblasts are the primary source of ECM [50,51], dynamic regulation of the EAM lineage provides a novel direction for targeted strategies that are aimed at delaying the development of chronic kidney disease and fibrosis.

### 5.2. CD38hi Macrophages

Nicotinamide adenine dinucleotide (NAD+) as an indispensable core coenzyme in cellular metabolism participates in metabolic pathways such as oxidative phosphorylation. NAD+ provides energy for vital physiological activities, effectively scavenges intracellular free radicals, and protects the structural and functional integrity of mitochondria. In addition, as a substrate for various enzymes, NAD+ is involved in DNA repair, maintains genomic stability, and delays cellular senescence [52]. CD38 is a transmembrane protein that is widely expressed in various immune cells that include T cells, B cells, and macrophages, with its primary function being to use NAD+ as a substrate and to catalyze its degradation into cyclic ADP-ribose, ADP-ribose, nicotinamide, and other products [53]. This catalytic process consumes substantial amounts of intracellular NAD+, thereby impairing physiological activities mediated by NAD+ such as cellular energy metabolism and DNA repair. NAD+ depletion also disrupts the dynamic equilibrium between ROS production and clearance, inducing oxidative stress imbalance and consequently accelerating cellular senescence. Senescent epithelial cells exhibit reduced regenerative capacity that directly promotes the fibrotic process [54]. In macrophages, CD38 expression can significantly enhance the secretion of inflammatory mediators such as IL-12 and IL-1β, thereby amplifying inflammatory responses at injury sites [55].

The inflammatory macrophage subtype characterized by high CD38 expression (CD38hi) exhibits the strongest pro-fibrotic effects, the severity of renal fibrosis is significantly correlated with the proportion of infiltrating CD38hi macrophages in kidney tissue [56], and the Csf1/Csf1r pathway serves as a key driver of their differentiation. Following CD38 gene deletion or treatment with CD38 inhibitors, both the degree of renal epithelial cell senescence and the severity of renal fibrosis are reduced. CD38-expressing CD68+ macrophages can be detected in human AKI and CKD renal tissues, and increased CD38 is associated with tubular injury and fibrosis in human samples [56]. Therefore, the development of targeted therapies against CD38hi macrophages (such as CD38 inhibitors and Csf1/Csf1r pathway blockers) or strategies for the selective elimination of CD38hi macrophages may represent potential future interventions for inhibiting the transition from AKI to CKD and delaying the progression of CKD.

### 5.3. Circulating M2 Macrophages

Thrombospondin-1 (THBS1) is primarily stored in platelet α-granules, and upon platelet activation by signals such as thrombin, collagen, and immune complexes, THBS1 is rapidly released. THBS1 subsequently binds to the platelet surface glycoprotein IIb–IIIa complex, forming a positive-secretion feedback loop that enhances platelet coagulation and inflammatory recruitment [57]. Previous studies have revealed that THBS1 participates in tissue fibrosis through mechanisms that principally involve the activation of downstream pro-fibrotic signaling pathways and the regulation of immune cell phenotypes [58].

Injury signals such as ischemia–reperfusion induce platelet activation, and the released THBS1 activates the TGF-β/Smad pathway, promoting M2 macrophage polarization, amplifying pro-fibrotic effects, and accelerating impairment of renal function [59]. Authors have observed reduced numbers of M2-like macrophages and attenuated renal fibrosis in ischemia–reperfusion mouse models treated with the THBS1 antagonist LSKL peptide or subjected to THBS1 gene deletion. THBS1-associated macrophages show partial human evidence. The presence of THBS1 in the plasma of patients with AKI was evaluated using RT-PCR methods [60]. These findings further confirm the role of the “platelet–THBS1–TGF-β/Smad–M2-like macrophage” pathway in promoting maladaptive repair following kidney injury.

### 5.4. S100a9-Dependent Inflammatory Macrophage Subsets

S100a9 is a Ca^2+^-binding protein that is primarily expressed in neutrophils and monocytes, and its primary functions involve the regulation of inflammatory processes and immune responses [61]. Inflammatory mediators such as TNF and IL-β induce abnormally high expression of S100a9, and S100a9 suppresses M2 macrophage expression, promoting macrophage polarization toward the M1 phenotype via activation of the TLR4/NF-κB pathway as it amplifies local inflammation and sustains the deterioration of an inflammatory microenvironment [62].

An infiltrating macrophage subset that is characterized by high expression of the S100a9 gene (S100a9hi) was identified in a mouse model of renal ischemia–reperfusion injury, with the subset representing the earliest responding, peripherally derived macrophages following injury and exhibiting the strongest pro-inflammatory activity [63]. During the early phase of injury, KRMs release pro-inflammatory chemokines such as CXCL1, CCL2, and CCL3—as well as induced S100a9 expression—thereby selectively recruiting the S100a9hiLy6Chi macrophage subset. These recruited cells activate the TLR4/NF-κB signaling pathway and release pro-inflammatory cytokines such as IL-1β and TNF-α; this process directly amplifies local inflammation and aggravates renal tissue injury while the cytokines further promote high S100a9 expression, forming a pro-inflammatory positive-feedback loop that continuously worsens the injury microenvironment. Administration of an S100a9 inhibitor has also been shown to reduce macrophage infiltration within tissues. The expression of S100a8/a9 significantly increased in the kidney tissues of AKI patients (nephrotoxicity or ischemic injury induced); urinary excretion of S100a8/a9 also increased in AKI patients, and the levels were significantly correlated with the severity of kidney tissue injury [63]. These findings highlight the critical role of S100a9hi macrophages in the initiation and amplification of inflammation during the early stages of kidney injury and provide crucial evidence for the subsequent development of persistent renal tissue damage and compromised repair.

### 5.5. Scar-Associated Macrophages

**Figure 2:**
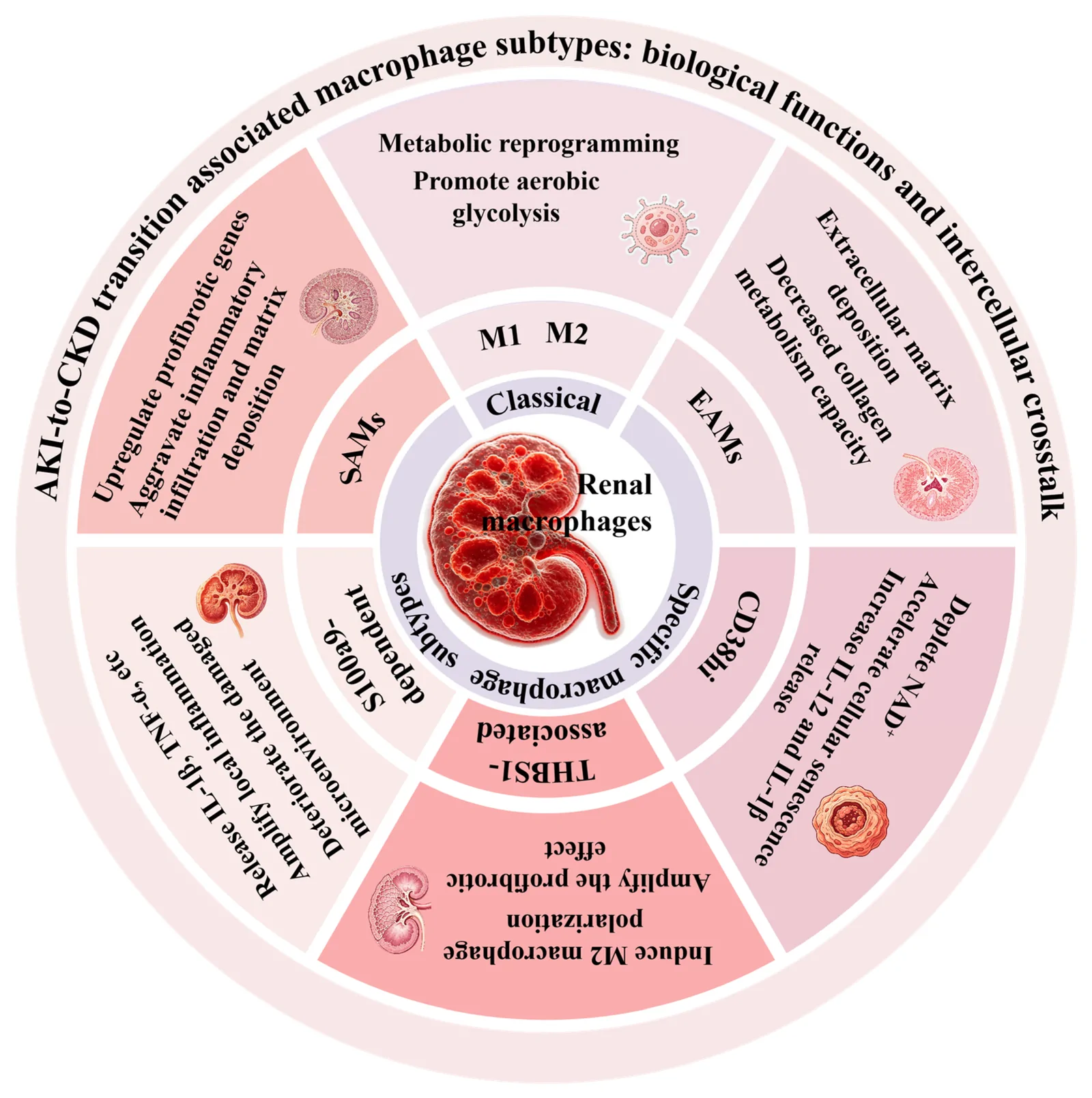
Macrophage Subtypes with Specialized Functions Observed During the AKI-to-CKD Transition. Macrophages of certain specialized functional subtypes engage in complex crosstalk with neighboring cells, forming a pathological signaling network that facilitates the progression from AKI-to-CKD. EAMs: Extracellular matrix remodeling-associated macrophages; CD38hi: high CD38 expression; THBS1: Thrombospondin-1; SAMs: Scar-associated macrophages. Created with WPS Office.

In 2019, Ramachandran and co-workers identified a novel macrophage subset within fibrotic scar regions of the liver using single-cell sequencing and formally defined it as scar-associated macrophages (SAMs) [64]. This macrophage subset was confirmed to be a key immune cell population that regulates the initiation and progression of organ fibrosis. Through in-depth investigations combining single-cell transcriptomic sequencing, clinical specimens, multiple animal models of kidney injury, and in vitro cellular experiments, Wang and associates [65] demonstrated that SAMs characterized by the augmented specific expression of Acp5 (TRAP5) gradually became the dominant macrophage population during the AKI-to-CKD transition and were enriched within fibrotic scar regions of the renal interstitium. TRAP5+ positive macrophages could be found in CKD patients kidney samples, whereas these cells were undetectable in AKI biopsies [65]. These findings indicate that this macrophage subset serves as a core effector population in the regulation of renal interstitial fibrosis. The specific regulatory mechanism underlying SAMs subset action involves extensive formation of secreted phosphoprotein 1 (SPP1) by injured renal tubular cells and interstitial fibroblasts. SPP1 specifically binds to the CD44 receptor on the surface of SAMs, activating them to enhance their pro-fibrotic biological activity while simultaneously inducing significant upregulation of intracellular TRAP5 expression. TRAP5, via its acid phosphatase activity, subsequently mediates the dephosphorylation of target proteins, activates the NF-κB signaling pathway, and upregulates the expression of pro-inflammatory and pro-fibrotic genes within SAMs—including TGF-β1, PDGF, CCL2, Spp1, Ctsk, and Mmp9—thereby intensifying inflammatory infiltration and abnormal matrix deposition in renal tissues. In addition, TRAP5 promotes the autocrine secretion of Spp1 by SAMs, forming a positive-feedback regulatory loop that stabilizes the pro-inflammatory and pro-fibrotic phenotype of SAMs [66,67]. These authors demonstrated that the targeted inhibition of TRAP5—thereby modulating SAM function—effectively reduced renal inflammatory infiltration, decreased abnormal extracellular matrix accumulation, and ultimately ameliorated renal fibrosis—blocking the transition from AKI to CKD. These findings confirm the premise that TRAP5 represents a reliable therapeutic target for the SAM-directed treatment of renal fibrosis (Figure 2).

It is worth noting that these macrophage subtypes are mainly identified in mouse ischemia–reperfusion injury models, and that human validation is still incomplete and warrants further investigation.

## 6. Therapeutic Strategies Targeting Macrophage Subtypes to Block the Transition from AKI to CKD

### 6.1. Inhibition of Inflammatory Macrophage Recruitment and Polarization

Targeting the recruitment and polarization of inflammatory macrophages represents a pivotal therapeutic strategy to interrupt the progression from AKI to CKD. One of the key mechanisms driving macrophage infiltration into injured renal tissue involves chemokine signaling, particularly through chemokine receptor 2 (CCR2). CCR2 antagonists have demonstrated efficacy in preclinical AKI models by blocking the mobilization of monocytes from the bone marrow to the kidney, thereby reducing the infiltration of pro-inflammatory M1-like macrophages [68]. For instance, in murine ischemia–reperfusion injury (IRI) models, CCR2 inhibition significantly decreased M1 macrophage accumulation and attenuated early renal damage, highlighting the potential of chemokine receptor blockade in modulating the inflammatory milieu post-AKI [68]. Beyond recruitment, the polarization of macrophages towards the M1 phenotype is orchestrated by intracellular signaling pathways such as NF-κB and STAT1. Pharmacological inhibition of these pathways has been shown to suppress M1 polarization, thereby diminishing the secretion of deleterious cytokines that exacerbate renal injury and fibrosis. For example, suppression of NF-κB and STAT1 signaling in macrophages reduces the inflammatory cascade, which in turn delays CKD progression by limiting maladaptive repair processes [69]. Furthermore, targeting upstream regulators such as interferon regulatory factor 4 (IRF4), which modulates macrophage polarization, can also influence macrophage behavior. Deletion of IRF4 in myeloid cells reduces macrophage recruitment and activation, leading to decreased fibrosis after ischemic injury, underscoring the therapeutic potential of modulating macrophage polarization at the transcriptional level [29]. Collectively, these findings emphasize that inhibiting inflammatory macrophage recruitment and M1 polarization through chemokine receptor antagonists and signaling pathway inhibitors can effectively mitigate early kidney injury and impede the transition to CKD.

### 6.2. Promotion of Reparative Macrophage Function and Resolution

Enhancing the reparative functions of macrophages and facilitating their resolution is a promising therapeutic avenue to promote kidney repair and prevent fibrosis following AKI. Reparative macrophages, often characterized by an M2-like phenotype, contribute to tissue remodeling by clearing apoptotic cells and secreting anti-inflammatory cytokines such as IL-10 and TGF-β in a controlled manner. Therapeutic strategies that augment M2 macrophage activity include administration of colony-stimulating factor 1 receptor (CSF1R) agonists or infusion of exogenous M2-like macrophages [70]. These interventions have been shown to enhance phagocytic clearance of dead cells and promote anti-inflammatory cytokine production, thereby improving renal repair outcomes in experimental AKI models. Moreover, metabolic reprogramming of macrophages via activation of PPARγ agonists, such as pioglitazone, has emerged as a novel approach to shift macrophages toward an anti-inflammatory and pro-repair phenotype. PPARγ activation enhances fatty acid oxidation in macrophages, which is associated with M2 polarization and reduced fibrogenesis. This metabolic modulation decreases the secretion of profibrotic factors and attenuates renal fibrosis, as demonstrated in preclinical studies [71]. The ability of PPARγ agonists to regulate macrophage metabolism and phenotype underscores the importance of cellular bioenergetics in macrophage function during kidney repair. Additionally, the timing of therapeutic intervention is critical; promoting M2 macrophage activity during the resolution phase of AKI can facilitate tissue regeneration while minimizing the risk of excessive fibrosis. These reparative strategies complement approaches aimed at suppressing inflammatory macrophages, together providing a balanced modulation of macrophage dynamics to optimize kidney recovery.

### 6.3. Interference with Macrophage Crosstalk Signaling

Disrupting the pathological crosstalk between macrophages and other renal cells is a critical strategy to prevent maladaptive repair and fibrosis during the AKI to CKD transition. Macrophages secrete profibrotic mediators such as TGF-β, which activate downstream Smad signaling pathways in fibroblasts and tubular epithelial cells, promoting extracellular matrix deposition and EMT. Targeting TGF-β signaling with neutralizing antibodies or small molecule inhibitors like galunisertib effectively blocks this fibrogenic communication, reducing fibrosis in experimental kidney injury models [72]. Furthermore, macrophage-derived extracellular vesicles (EVs) containing miRNAs such as miR-21 and miR-214 have been implicated in modulating tubular epithelial cell apoptosis and mitochondrial autophagy, processes that contribute to maladaptive repair and fibrosis [73,74]. Therapeutic strategies aimed at inhibiting the release or uptake of these EV-associated miRNAs can modulate epithelial cell fate and mitochondrial function, offering novel targets for intervention. For example, suppression of miR-384-5p has been shown to protect tubular epithelial cells and reduce fibrosis [75].

Among these therapeutic candidates, CCR2 antagonist CCX140-B had entered clinical trials for diabetic kidney disease and focal segmental glomerulosclerosis [76,77], yet no clinical trial has been specifically designed for AKI-triggered renal fibrosis. Results from completed clinical trials of CCR2 inhibitor DMX-200, dual CCR2/CCR5 antagonist PF-04634817 and CCL2 neutralizer emapticap pegol reveal that these agents modestly ameliorated renal injury-related abnormalities such as proteinuria and elevated serum creatinine, yet therapeutic efficacy remains suboptimal, and most candidates fail to advance into phase III investigations [78,79,80]. Other strategies including NF-κB/STAT1 inhibitors, CSF1R agonists and miRNA-targeting interventions remain largely at the pre-clinical stage for renal-fibrosis indications. Their efficacy for treating post-AKI renal fibrosis still lacks human evidence and further validation is required.

## 7. Conclusions

Several targeted therapeutics have been clinically applied for kidney diseases, such as the SGLT2 inhibitor dapagliflozin, selective nonsteroidal mineralocorticoid receptor antagonist finerenone, and multiple immunotargeted agents indicated for specific glomerulopathies. Extensive evidence indicates that advanced-stage CKD are universally accompanied by widespread renal fibrosis. Inflammatory responses serve as a critical driving force of fibrotic progression. Therefore, macrophages, the principal innate immune cells orchestrating intrarenal inflammatory networks, together with their downstream effector molecules, represent promising therapeutic targets and offer innovative strategies for the treatment of kidney diseases.

Advances in single-cell RNA sequencing and spatial transcriptomics enable the precise identification of functionally specialized macrophage subsets involved in renal disease progression. Mounting preclinical studies demonstrate that targeted modulation of macrophage recruitment, activation, phenotypic polarization and effector functions confers protective effects against kidney damage. Nevertheless, these therapeutic approaches are solely supported by preclinical animal data, and their clinical benefits remain to be validated in clinical trials.

Future studies should prioritize spatial transcriptomic and spatial proteomic analyses using human renal biopsy samples, to dissect the molecular signatures and spatial localization of macrophage subsets in kidney tissues from AKI and CKD patients. These efforts will facilitate the construction of comprehensive human renal macrophage atlas and systematically delineate divergent functional characteristics of distinct macrophage subsets under physiological conditions and during the pathological progression of AKI-to-CKD transition. Furthermore, exploring subset-specific biomarkers derived from pathogenic macrophages may provide potential evidence for early identification and stratified intervention of high-risk AKI patients. In parallel, it is necessary to further decipher the dynamic regulatory patterns of macrophage immunometabolic reprogramming at different injury stages, so as to identify druggable targets capable of reversing the profibrotic phenotype of macrophages.

## Data Availability

No new data were created or analyzed in this study. Data sharing is not applicable to this article.
